# Second Childhood

**Published:** 1895-06

**Authors:** 


Second Childhood.—Columbus, Ind., Feb. 27.—Mrs. Ann Featherstone, aged ninety-eight, has been ill for three weeks. Her physician to-day announced his patient as convalescent, she having successfully cut a large jaw tooth.

